# Estimation of Quality‐adjusted Life Years alongside clinical trials: the impact of ‘time‐effects’ on trial results

**DOI:** 10.1111/jphs.12218

**Published:** 2018-02-26

**Authors:** Morro M.L. Touray

**Affiliations:** ^1^ University of Surrey Guildford UK; ^2^ University of South Wales Pontypridd UK

**Keywords:** health economics, outcome research, patient satisfaction, quality‐adjusted life years

## Abstract

**Objectives:**

The objectives were to investigate the impact of ‘time‐effect’ on the estimation of quality‐adjusted life years (QALYs) along prospective clinical trials’ outcomes using an assumed fixed time duration versus the actual time durations for each case. The ‘time’ duration is the length of time in a health state.

**Methods:**

Two methods were used in the estimation of QALYs based using EQ‐5D 3L scores collected at specific time‐point intervals. One method used the actual time durations for each case based on CRF records, and the other used an assumed time duration and globally applied it to all the cases. Using SPSS ^®^ software program, we used paired‐sample *t*‐tests to assess whether the ‘time‐effect’ can potentially affect trial results using CONSTRUCT trial data as reported in the trial results publications. The trial compared use of Infliximab with Cyclosporine for patients with Ulcerative Colitis and it involved some 270 participants.

**Key findings:**

The results largely indicate statistically significant differences between the two methods of QALY estimations. QALYs at the respective time‐points indicate no statistical difference between the two approaches. However, the difference in terms of total QALYs between the two QALY estimation approaches is statistically significant with considerable impact on costs/QALY.

**Conclusions:**

Considering the possible impact of the time‐effect on QALY estimations, the result implies that it can have significant implications for resources allocations decisions. In this respect, researchers have to pay due considerations to the approach they use and where possible, actual time durations must be used in QALY estimations along prospective clinical trials.

## Introduction

EQ‐5D‐3L is a generic preference‐based patient‐reported outcome measure which can be used to provide estimations of quality‐adjusted life years (QALYs) gained. QALYs is a measure of quality and quantity of life (i.e. a product of the health‐related utility state and the number of times or period in that state). Estimations of QALYs from EQ‐5D utility scores collected at different time‐points along clinical trials can be made using two approaches. The two approaches are the application of the same fixed period of time between visits for all participants, for example 3 months (converted to years), if data were supposed to be collected at 3‐month interval for participants or using the actual period between visits using the actual dates when the EQ‐5D‐3L data were collected as recorded on Case Report Forms (CRFs) or Patient Follow‐up Questionnaires (PFQs) in the estimation of QALYs. In the second method, the time period would be the difference between the recorded dates on the CRFs or PFQs. For example, the period/time (in years) between two time‐points for CRFs/PFQs with dates 05/10/2012 and 21/01/2013 would be 0.29589 assuming 1 year to be 365 days.

The objective of this study was to evaluate the possible impacts of the two methods of QALY estimation along clinical trials on the trial results using patient‐level data from the CONSTRUCT clinical trial. The null hypothesis for this research is that there is no statistically significant difference of means between total QALYs for the fixed‐period method and the non‐fixed‐period approaches (i.e. the one for which the periods or time factors for QALY calculations are based on actual EQ‐5D dates as provided on the CRFs and PFQs). While total QALYs are the main outcome of interest for this study, differences between the two approaches for QALYs at the respective data collection points are also examined.

The CONSTRUCT trial was a non‐blinded controlled study which compared Infliximab with Cyclosporine for patients with Ulcerative Colitis.^1^ Infliximab and Cyclosporine are among few immunosuppressive drugs which are predominantly in use for treatment of steroid‐resistant Ulcerative Colitis.^1^, ^2^ The trial was funded by National Institute for Health Technology Assessment Programme and conducted by the College of Medicine, Swansea University. The economic evaluation component was subcontracted to the Health Economics and Policy Research Unit, University of South Wales. The protocol and the results of the trial have been reported by Seagrove *et al*.^1^ and Williams *et al*.^3^, ^4^ respectively.

## Methods

**Figure 1:**
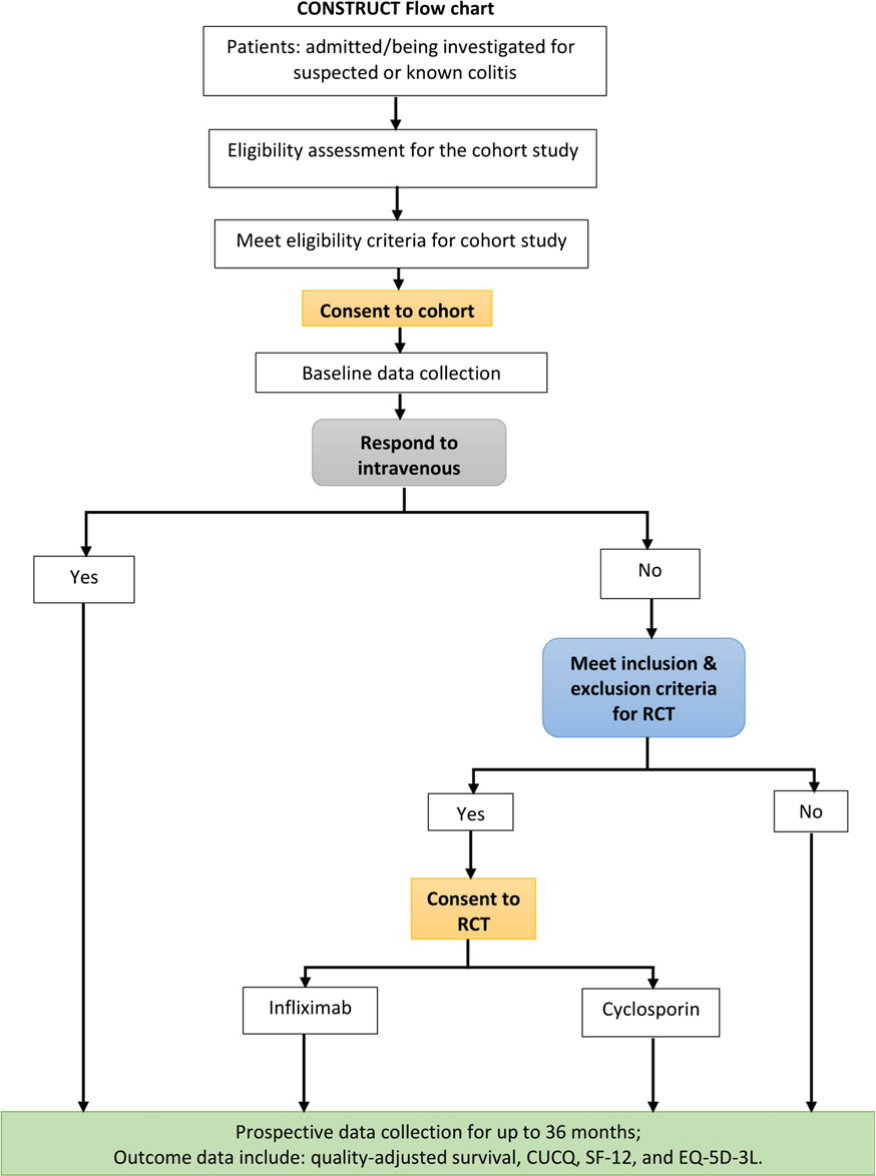
CONSTRUCT trial flow chart adopted from trial protocol (Seagrove *et al*.^1^).

Comprehensive description of the trial design has been provided in Seagrove *et al*. paper. Figure 1 provides the flow chart of the clinical trial.

Participants were randomly allocated to either Infliximab or Cyclosporine in equal proportions. In all, 52 UK centres (including large teaching hospitals) across various NHS Health Boards or Trusts in Wales, England and Scotland, participated in the study.^1^

The main clinical outcomes (results of which have been reported by Williams *et al*.^3^, ^4^ include mortality, colectomies, readmissions, quality of life and cost‐effectiveness of the interventions. Data on these measures were collected from participants at baseline, 3, 6, 12, 18, 24, 30 and 36 months. The target population were patients with acute severe Ulcerative Colitis who failed to respond to 2–5 days course of intravenous steroid treatment and did not need surgery. Of the 270 randomised to the clinical trial 100 were female and 250 being White.^3^, ^4^ The mean weight and height were 74.14 kg and 1.71 m respectively. One hundred and twelve indicated that they have never smoked.^3^, ^4^

The quality of life outcomes were measured using EQ‐5D‐3L, CUCQ and SF‐6D. The EQ‐5D‐3L health state values were used in the estimation of QALYs. The quality of life instruments were completed by participants at specific time intervals cited earlier.^1^ These intervals (or data collection time‐points) are key components in the estimation of QALYs.

The estimation of QALYs adopted a linear approach based on Manca *et al*.^5^ as in the formula below.

$$\begin{array}{cc} & \mathrm{Total}\;\mathrm{QALYs}\\ & =\frac{\;x_{1}\;+\;x_{2}}{2}\times p_{1}+\frac{x_{2}+x_{3}}{2}\times p_{2}\;+\frac{x_{3}+x_{4}}{2}\times p_{3}\;+\frac{x_{4}+x_{5}\;}{2}\times p_{4} \end{array}$$

where *x*_i_ represents EQ‐5D‐3L utility scores at respective data collection points and *p*_i_, the periods between the EQ‐5D‐3L utility scores for the respective data collection points. This assumes a linear relationship between data collection time‐points and can be described as the area under the straight lines. The periods between visits can be calculated using patient‐level data using the dates recorded on the respective CRFs and PFQs (i.e. on the completed EQ‐5D‐3L forms).

In the application of this formula, two methods were used. One was the use of the same equal periods of time between data collection points of baseline, 3, 6, 12 months and the like across for all cases as applicable, and the other is the use of actual dates (reflecting when the scores were provided) on the questionnaires to calculate the time periods in the estimation of QALYs.

Where there were cases of missing data for EQ‐5D‐3L utility values, data for the last/previous known state are used consistent with White *et al*. (2011).^6^ Participants lost to follow‐up for any particular reason including deaths and withdrawals were not included in EQ‐5D utility analyses. Participants with only one EQ‐5D records were as well not included in any estimation of QALYs.

In the case of missing dates, if it is between two data collection points, the mid‐point between the dates is used. If it is at the end, for example when we have dates for the 30‐months’ data collection point but not for the 36‐months’ collection point, then 182 days which is supposed to be the period in days between the 30th and 36th months is added to compute the missing date. If a date is provided but not EQ‐5D‐3L utility value, then it is treated as a missing value. Where two EQ‐5D‐3L questionnaires are recorded for a participant and the period between them, span over many data collection points then the intervening data collection points is treated as missing and not as if the participant has only two EQ‐5D utility values.

As this study is intended to highlight essential methodological issues in the calculation of QALYs along clinical trials, it will not be reporting or commenting on the final results of the CONSTRUCT trial. Central to the analyses for this research is ‘total QALYs’ in the two QALY estimation approaches (i.e. using fixed and non‐fixed time periods in the estimation of QALYs). In these, both discounted and non‐discounted total QALYs were examined. A discount rate of 3% was used. Even though total QALYs are the main outcome of interest for the comparison of the two approaches, QALYs at the respective data collection points, that is, at 3, 6, 12, 18, 24, 30 and 36 months using the two approaches were analysed as well. Paired‐sample *t*‐test was used to compare the two approaches with and without bootstrapping using total QALYs.

To test the statistical difference between two measurements that are from the same sample, paired‐sample *t*‐tests also known as repeated measures *t*‐tests were performed. Prior to conducting this test, the data were examined to ensure that all the essential conditions for the test, such as assumptions of normality, issues of outliers and the dependent variable to be a continuous data among others, are met. Had these conditions not been met it would have been appropriate to use Wilcoxon signed‐ranks test instead, which is a nonparametric test used where data are not assumed to be normally distributed.

Paired‐sample *t*‐tests were performed on both QALYs estimated at the respective data collection time‐points, and the main outcome of interest which is total QALYs (discounted and non‐discounted) estimated using the two approaches being investigated. These are use of fixed time periods and patient‐level data dates in the calculation of QALYs. Total QALYs are the main outcome of interest for reasons that it is the total QALYs that determine the final HRQoL results of a clinical trial and therefore feed into UK's National Institute of Health and Care Excellence's (NICE) threshold considerations for the use of a drug or otherwise in the National Health Service. Bootstrapping was also used to assess whether differences between the two approaches will continue to be significant even with resampling or data replication.

All the analyses were performed using SPSS^®^ of IBM Corporations, Armonk, NY, USA. The mapping of EQ‐5D‐3L scores to utility values was performed using SPSS^®^. The same program was used to organise the data as well as for the handling of missing data.

## Results

In all, 270 participants with acute severe Ulcerative Colitis who failed to respond to intravenous steroid and did not need surgery were randomised to participate in the two‐arm non‐blinded clinical trial. Of this, 12 participants withdrew consent for both CRF and PFQ and were therefore not included in any of the analyses in any way or form hence 258 participants remained for inclusion in the analyses. Table 1 provides details of the number of available EQ‐5D‐3L data used in the estimation of QALYs at the respective data collection points.

**Table 1:** EQ5D data available at various collection points for estimation of QALYs

		Frequency	Percentage
EQ5D@3M	Not included	25	9.3
	Included	224	83.0
	Not include_1_EQ5D	21	7.8
	Total	270	100.0
EQ5D@6M	Not included	25	9.3
	Included	224	83.0
	Not include_1_EQ5D	21	7.8
	Total	270	100.0
EQ5D@12M	Not included	29	10.7
	Included	220	81.5
	Not include_1_EQ5D	21	7.8
	Total	270	100.0
EQ5D@18M	Not included	74	27.4
	Included	175	64.8
	Not include_1_EQ5D	21	7.8
	Total	270	100.0
EQ5D@24M	Not included	130	48.1
	Included	119	44.1
	Not include_1_EQ5D	21	7.8
	Total	270	100.0
EQ5D@30M	Not included	177	65.6
	Included	72	26.7
	Not include_1_EQ5D	21	7.8
	Total	270	100.0
EQ5D@36	Not included	237	87.8
	Included	33	12.2
	Total	270	100.0

QALYs, quality‐adjusted life years.

### Paired‐sample *t*‐tests

The analyses of QALYs for the various data collection time‐points largely indicate no significant differences between the two methods at the respective time‐points (Table 2). The only place where there is significant difference is at 6th Months’ time‐point where the 2‐tailed significant *P*‐value is 0.005.

**Table 2:** Paired‐sample *t*‐tests for quality‐adjusted life years (QALYs) for the respective data collection points

Paired samples test										
		N	Paired differences					*t*	df	Sig. (2‐tailed)
			Mean	Std. deviation	Std. error mean	95% confidence interval of the difference				
						Lower	Upper			
Pair 1	QALY_3M – QALY_3M_Fixed	224	0.0030837	0.0379836	0.0025379	−0.0019176	0.0080850	1.215	223	0.226
Pair 2	QALY_6M – QALY_6M_Fixed	224	0.0106664	0.0562696	0.0037597	0.0032573	0.0180754	2.837	223	0.005
Pair 3	QALY_12M – QALY_12M_Fixed	220	0.0026320	0.0856848	0.0057769	−0.0087534	0.0140173	0.456	219	0.649
Pair 4	QALY_18M – QALY_18M_Fixed	175	0.0008077	0.0687918	0.0052002	−0.0094559	0.0110712	0.155	174	0.877
Pair 5	QALY_24M – QALY_24M_Fixed	119	0.0145399	0.0991509	0.0090892	−0.0034591	0.0325389	1.600	118	0.112
Pair 6	QALY_30M – QALY_30M_Fixed	72	−0.0031073	0.0916555	0.0108017	−0.0246453	0.0184307	−0.288	71	0.774
Pair 7	QALY_36M – QALY_36M_Fixed	33	−0.0092164	0.1071548	0.0186532	−0.0472118	0.0287790	−0.494	32	0.625

With regard to the main outcome of interest, total QALYs, there is statistically significant difference between the two QALY calculation approaches. Even after conducting bootstrapping, the 2‐tailed significance *P*‐values on the differences between the two approaches remained significant for both discounted and non‐discounted total QALYs. The bootstrapping results are based on 10 000 bootstrap samples. Table 3 provides details on the results of paired‐sample tests on total QALYs for the two methods. These results demonstrate the potential impact of ‘time‐effect’ on the outcome of QALY estimations along clinical trials for EQ‐5D scores collected at different time‐points.

**Table 3:** Paired‐sample *t*‐test and bootstrap for paired sample *t*‐test on total quality‐adjusted life years (QALYs)

		Paired‐sample test									Bootstrap for paired‐sample test				
		Paired differences						*t*	df	Sig. (2‐tailed)	Bootstrap^a^				
		N	Mean	Std. deviation	Std. error mean	95% Confidence interval of the difference					Bias	Std. error	Sig. (2‐tailed)	95% confidence interval	
						Lower	Upper							Lower	Upper
Pair 1	QALY_Total – QALY_Total_Fixed	224	0.0223393	0.0969739	0.0064793	0.0095707	0.0351079	3.448	223	0.001	0.0000440	0.0064722	0.001	0.0101480	0.0353085
Pair 2	QALY_Total_Disc – QALY_Total_Fixed_Disc	224	0.0148629	0.0642777	0.0042947	0.0063995	0.0233264	3.461	223	0.001	−0.0000844	0.0043089	0.001	0.0062421	0.0232129

a Unless otherwise noted, bootstrap results are based on 10 000 bootstrap samples.

The cost‐effectiveness analysis in Williams *et al*.^3^, ^4^ indicated mean cost per QALY to be £7604.89 and £10 664.38 for Cyclosporine and Infliximab, respectively, over 30‐month period. These costs were weighted by participants’ time in the clinical trial. As this study covers a period of 36 months, we have made the assumption that above costs over 30 months should not significantly change in the additional 6‐month period. In this respect, applying the above mean costs would result in cost‐effectiveness ratio of £93504.09/QALY using actual time period approach in calculation of QALYs. When the fixed‐period approach is used, the cost‐effectiveness ratio has been increased to £119575.83/QALY in favour of Cyclosporine.

## Discussions

Based on the results, the paper emphasises the need for researchers to report the approach they employed in their estimation of QALYs from EQ‐5D utility data collected at different time‐points along clinical trials due to its possible implications on trial results. These results have consistently indicated significant differences between the outcomes of the two approaches even with data replication. The application of the mean costs to these results also indicates stark differences between the two approaches.

Patient‐reported outcome measures (PROMs) proved to be vital in providing yardsticks in many areas of healthcare delivery around the world. While there can be many reasons for this, the main thing is that it is because patients themselves provide the information. In this light, PROMs provide validated evidences of health from the patients’ point of view hence can be used to assess health needs and levels in populations particularly the users of healthcare services. PROMs can therefore serve as evidences of the impact and outcomes of healthcare interventions and services.

EQ‐5D is one of the non‐disease‐specific measurements (generic instruments) used in this research, which feeds into HRQoL measures used by researchers and decision makers for healthcare analysis and resources allocations amongst others. In the context of the United Kingdom, EQ‐5D is one of those preference‐based measurements used in the estimation of QALYs, which is mathematically a product of the health utility and the time spent in that health utility. QALYs are key yardsticks in health economic decisions regarding the introduction of healthcare interventions into the National Health Service.

The implications of these current research results with respect to the differences between the two QALY calculation approaches along clinical trials and for that matter the potential impact of ‘time‐effect’ on total QALY outcome can be essential for many reasons. First, EQ‐5D scores reported by a patient are not a retrospective reflection of the person's health state but the health state at the ‘time’. So using a different date and for that matter ‘time’ is a misrepresentation of realities. Second, in real‐life situations, patients should not be expected to be reporting at exactly the same time intervals. In addition to appointment failures, there are also variations in terms of waiting time across practices. Third, studies that may not have the time and resources to conduct RCTs, such as those using meta‐analysis, may be using mean total QALY results of these types of trials to feed into their research. The outcomes of clinical trials can therefore greatly influence the results of such meta‐analyses studies. Fourth, as indicated in the costing and cost‐effectiveness projections, it is clear that it can have resources allocation decision implications. The increment in terms of cost‐effectiveness ratios between the two approaches is well over 20% which can be fundamental considering limited healthcare resources challenges.

This analysis has a number of limitations. The sample size can be considered to be too small. Because of the small sample size bootstrapping was used which is a standard practice in economic evaluation analysis conducted alongside clinical trials.^7^, ^8^, ^9^, ^10^ As a result of the small sample size, the mean difference in terms of days between the two approaches is about 8 days. However, 8 days can have enormous healthcare resources implications as demonstrated in the economic evaluation projections. The missing data imputation approaches used in Williams *et al*.^3^, ^4^ and this study differ. This explains the differences in terms of numbers (for the respective data collection points) and QALYs gained in the descriptive analysis of this study and that of Williams *et al*.^3^, ^4^

Differences between QALY totals at most of the data collection points are not statistically significant (see table). However, the hypothesis for this analysis is premised on the overall QALYs’ total and not that of the respective data collection points. It is not these individual QALYs at the specific data collection points along a prospective trial which represent the final QALY outcome. It is instead the total QALYs (which is the sum of the QALYs estimated at the various time‐points).

Considering the statistically significant differences between the two methods as revealed by this current analysis, it is imperative and essential for researchers to report which of the two approaches they employed in the estimation of QALYs from preference‐based utilities collected at different time‐points along clinical trials. It is also vital that they report confidence intervals for such mean scores to inform possible sensitivity analysis to be used in other studies that may be using their results. So far, there are no evidences indicating that researchers are providing information on the approaches they used with regard to the ‘time‐effect’ in the calculation of QALYs from preference‐based utility values collected at different time‐points along clinical trials.

## Conclusion

Quality‐adjusted life years are prominent yardsticks in economic evaluation of healthcare interventions in the United Kingdom. QALYs are in fact the recommended measure of disease burden by NICE.^11^ The most frequently used health‐related quality of life instrument in the estimation of QALYs has been EuroQol's EQ‐5D instrument.^12^ The estimation of QALYs in this prospective clinical trial was based on EQ‐5D‐3L which was collected at specific time‐points.

Quality‐adjusted life years are a product of health utilities and the ‘time’ a person spent in that health utilities state. This therefore explains the possible impact the ‘time‐effect’ can have on QALY estimations along prospective clinical trials where utility values are collected at specific time‐points.

That time component in QALY estimations can be calculated using the exact dates on patient records, that is the dates the utility values were taken or just using the same assumed time for all the participants. Using paired‐sample *t*‐test, this study reveals statistically significant difference between the two approaches demonstrating the potential impact the ‘time‐effect’ can therefore have on trial results both in terms of QALYs and the attendant cost‐effectiveness. The cost‐effectiveness ratio was £93 504.09/QALY using actual time period approach in calculation of QALYs. When the fixed‐period approach was used, the cost‐effectiveness ratio increased to £119 575.83/QALY. This result does not only justify the need for researchers to pay due considerations to the approach they used in QALY estimations, but most importantly, it emphasises the need for researchers to use actual time durations in QALY estimations along prospective clinical trials.

## Declarations

### Conflict of interest

The Authors declare that they have no conflicts of interest to disclose.

### Funding

This research received no specific grant from any funding agency in the public, commercial or not‐for‐profit sectors.

### Author contributions

All Authors state that they had complete access to the study data that support the publication.
